# Cell-Free Fetal Deoxyribonucleic Acid (cffDNA) Analysis as a Remarkable Method of Non-Invasive Prenatal Screening

**DOI:** 10.7759/cureus.29965

**Published:** 2022-10-05

**Authors:** Himanshu Raj, Pallavi Yelne

**Affiliations:** 1 Medicine, Jawaharlal Nehru Medical College, Datta Meghe Institute of Medical Sciences, Wardha, IND

**Keywords:** cell-free dna, sensitivity, non-invasive, trisomy, down syndrome, chromosome, antenatal screening

## Abstract

The cell-free fetal DNA (cffDNA) analysis for screening fetal genetic anomalies has increased dramatically since its commercialization in 2011 worldwide. In the early weeks of pregnancy, it offers a hassle-free, non-invasive procedure of antenatal screening. It guides and protects mothers from undergoing unwanted risk-laden invasive prenatal testing. cffDNA testing is accurate at detecting the abnormal fetus chromosome among a large pool population. Patau syndrome, Edward syndrome, and Down syndrome are currently being accurately screened by this method. Due to their sensitivity and specificity, they now have become the screening method of choice, approaching almost 100% in various studies with a large sample pool. The latest procedures to analyze cffDNA, like the new digital droplet polymerase chain reaction (ddPCR) and sophisticated next-generation sequencing (NGS), have increased detection rates with decreased analyzing time. The latest techniques make it possible to screen large numbers of the population with faster report generation. Screening for Rh incompatibility and its timely prevention is now more accessible and more accurate with the help of cffDNA analysis. The problem arises when we deviate from the primary disease and start testing for anomalies not intended to be screened by cffDNA in the first place. Fetal sex chromosome aneuploidy screening by cffDNA is one area where the test gives mixed results either due to differences in machinery, laboratory parameters, or human error. Other rare occurrences like trisomes, such as trisomy 7, trisomy 16, trisomy 22, and a few microdeletion syndromes are also being screened but with less accuracy. Like every technology, cffDNA analysis is not entirely free of criticism. Its high testing cost, potential to accurately prognosticate the gender of the developing fetus and absence of standard testing practices will become an issue as the test becomes routine worldwide.

## Introduction and background

**Figure 1:**
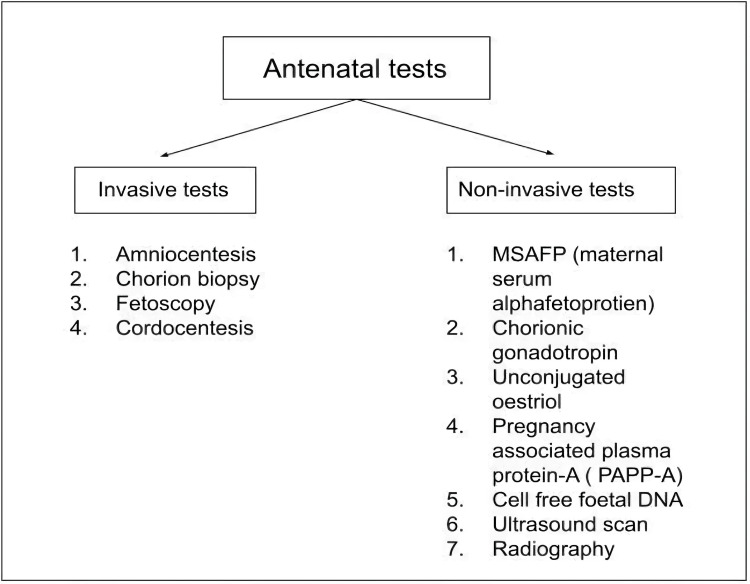
Classification of Antenatal tests. MSAFP: Maternal serum alpha-fetoprotien, PAPP-A: Pregnancy associated plasma protien-A, DNA: Deoxyribonucleic acid [4]

Out of 65,797 babies born in India every day, almost 81 babies are born with a common chromosomal defect known as Down syndrome, with the prevalence being one out of every 803 live births [1,2]. This number grows to 405, with prevalence being one out of 166 live births when all genetic abnormalities are considered. Most of these chromosomal defects will cause mental retardation, sex anomalies or congenital anomalies in live-born babies. Of all clinically recognized pregnancies, around 10% resulted in miscarriage, of which half of early pregnancy losses are due to genetic abnormalities [3]. Hence most parents opt for antenatal screening of the fetus, which has now become a routine investigation for all pregnant females in this modern world. Antenatal screening is defined as procedures performed during different antenatal periods to detect health problems of the fetus or diagnose conditions related to the mother which will affect the expected growth of the fetus. We have multiple screening modalities to screen an antenatal mother efficiently and precisely. Antenatal testing has been broadly divided into invasive tests and non-invasive testing, as shown in Figure 1. 

First performed in 1956 for the diagnosis of a common genetic disease, amniocentesis was the first invasive test to be carried out to detect chromosomal abnormalities, especially Down syndrome (trisomy 21), through karyotyping amniotic fluid cells [5]. With time, the tests became more refined such that most of the non-invasive procedures are regarded as diagnostic due to their 100% sensitivity. Besides confirming a diagnosis by performing several tests from the tissue sample obtained, most of these tests also carry a potentially significant risk to maternal and fetal health by putting the pregnancy at risk. As mentioned in Table 1, procedures like amniocentesis and chorionic villi sampling involve creating communication between the fetus and the external environment. Invasive procedures predispose an otherwise normal pregnancy to certain complications, which include chances of miscarriage, bleeding, infection, harm to the fetus from needle penetration, and rhesus disease. 

**Table 1:** Invasive prenatal testing procedures and their associated risks. [6,7]

Test	Associated risk
Amniocentesis	Chances of fetal loss: 0.13%, animatic fluid leakage: 1%-2%,
Chorionic villus sampling	Limb reduction defects, intrauterine infection, membrane rupture, fetal loss
Cordocentesis	The highest chance of fetal loss: 1%-3% (attributable risk)

NIPT is the method that detects the possibility of having genetic abnormalities or chromosomal disorders in a fetus. Non-invasive prenatal testing can only see a genetic condition's high and low risk. NIPT has an accuracy greater than 99 percent, but it is still a screening test, and it needs to be confirmed with the help of other diagnostic tests mentioned before, namely chorionic villi sampling and amniotic fluid test or amniocentesis. These are considered diagnostic because of the further tests like karyotyping or QF-PCR performed on these tissues, which yield diagnosis with 100% accuracy. Hence, with new and better non-invasive procedures, it is possible to screen the fetus accurately without putting the pregnancy at risk. Non-invasive procedures carry the least risk, which is associated with invasive testing. It also gives parents a sense of reassurance as the method of collecting samples for the test is easy to perform and almost painless. A summary of relevant prenatal screening tests is mentioned in Table 2. In all those methods, testing for cf-DNA (cell-free DNA) has recently emerged as the most promising non-invasive prenatal testing modality. 

**Table 2:** Summary of invasive and non-invasive prenatal tests. Rh: Rhesus factor, L/S ratio: Lecithin/ Sphingomyelin ratio, TORCH infections: Toxoplasmosis, Other (syphilis, varicella-zoster, parvovirus B19), Rubella, Cytomegalovirus and Herpes infections, MSAFP: Maternal serum alpha-fetoprotein, PAPP-A: Pregnancy-associated plasma protein A, IUGR: Intrauterine growth restriction, SGA: Small for gestational age, cffDNA: Cell-free foetal DNA, USG: Ultrasonography, CRL: Crown-rump length, MSD: Musculoskeletal disorders.

References	Name	Testing sample	Timing of test from	Abnormality screened/ diagnosed/ evaluated
Jindal, Sharma and Chaudhary. [5]	Amniocentesis	Amniotic fluid	15 weeks gestation to delivery	Sex-linked disorders, Rh isoimmunisation, inborn errors of metabolism, L/S ratio, TORCH infections, neural tube defects
Jindal et al. [5]	Chorion biopsy	Tissue aspirate of chorionic villi	10 to 14 weeks of gestation	Genetic diseases like Down syndrome, cystic fibrosis, sickle cell anaemia and sex-linked disorders
Deka et al. [8]	Fetoscopy	Foetal blood or tissue sample	17 to 20 weeks of gestation	Sickle cell anaemia, haemophilia, Tay Sachs disease, neural tube defects
Jindal et al. [5], Peddi et al. [9]	Cordocentesis	Foetal blood from umbilical cord	20 to 28 weeks of gestation	Haematological, inborn infectious disease, histopathologic analysis, metabolic assay, intrauterine growth retardation
Antsaklis et al. [10]	MSAFP	Maternal blood	14 weeks to 32 weeks of gestation	Neural tube defects, omphalocele, gastroschisis, down syndrome
Shiefa et al. [11]	Chorionic gonadotropin	Maternal urine or blood sample	8-10 weeks of gestation to delivery	Confirmation of pregnancy, ectopic pregnancy, trophoblastic disease, placental site trophoblastic disease
Betz and Fane. [12]	Unconjugated oestriol	Maternal blood serum and plasma	12 weeks of gestation till term	Trisomy 21, trisomy 18, foetal growth restriction, foetal demise, pregnancy loss, predicting the onset of labour
Shiefa et al. [11], Fruscalzo et al. [13]	PAPP-A	Maternal blood serum	10 to 14 weeks of gestation	Down syndrome, stillbirth, IUGR, SGA, preeclampsia
Pös, Budiš and Szemes. [14], Bedei et al. [15]	cffDNA	Maternal blood	Ten weeks of gestation till delivery	Down syndrome, Edward syndrome, Patau syndrome, haemoglobinopathies, phenylketonuria, Rh status
Ulrich and Dewald. [16]	USG	NIL	10-13 weeks of gestation till delivery	Foetal viability, CRL, MSD, gross foetal abnormality, chromosomal abnormalities, Intertwin membrane, placental abnormality
	Radiography	NIL	14 weeks of gestation till delivery	Diagnosis of pregnancy, foetal maturity, pelvimetry, congenital malformations

## Review

Cell-free fetal DNA 

Our blood contains fragments of our DNA, known as cell-free DNA or cfDNA. It refers to all non-encapsulated DNA present in the blood, and a portion of that cell-free DNA can originate from a tumor clone in the body [17]. It is called circulating tumor DNA or ctDNA. When the woman is pregnant, her blood will also contain some fragments of placental DNA, which has a genetic makeup identical to that of the fetus. By analyzing these parts of the DNA, we can determine if a person has an increased risk of having any tumor or, in the case of a pregnant female, an increased or decreased risk of certain genetic abnormalities that might affect the growing fetus. Non-invasive prenatal testing or screening: NIPT or NIPS, for short, was announced as a blessing to women when it was first inaugurated commercially in India in 2012. A test that includes taking just one sample of a vial of blood, is able to advise expecting parents whether their unborn child is suffering from any genetic anomaly that might render the child physically and mentally handicapped upon birth or even risk their life with 99% accuracy.

Diseases Screened by Cell-Free Fetal DNA

A cfDNA or cffDNA (cell-free fetal DNA) screening is used to screen whether the fetus is under an increased likelihood of the disorders related to its chromosomes. Non-invasive prenatal tests essentially look for an additional or misplaced copy of chromosome 23 (X) or chromosome Y known as gender-determining chromosomes. Additionally, it is also used to screen for particular other genetic abnormalities such as trisomy 18, which is caused by an extra chromosome 18, trisomy 13, caused due to an additional chromosome 13, trisomy 21, caused by an extra chromosome 21, Cystic fibrosis, Thalassemia, Phenylketonuria, Sickle Cell Anaemia or determine a baby's gender [18,19]. Determination of the gender may be done if an ultrasound scan is unable to establish whether the growing fetus is a male or female. A disorder of the sex chromosomes may cause this. cffDNA is also used to check Rhesus (Rh) blood type. If an Rh-positive fetus is developing inside the womb of an Rh-negative mother, the mother's body's immune system will attack the fetal blood cells. If the doctor finds out that the baby is Rh-positive ahead of time in pregnancy, they can take the proper interventions necessary to protect the fetus from threatening complexities.

The Procedure of cffDNA Analysis

**Figure 2:**
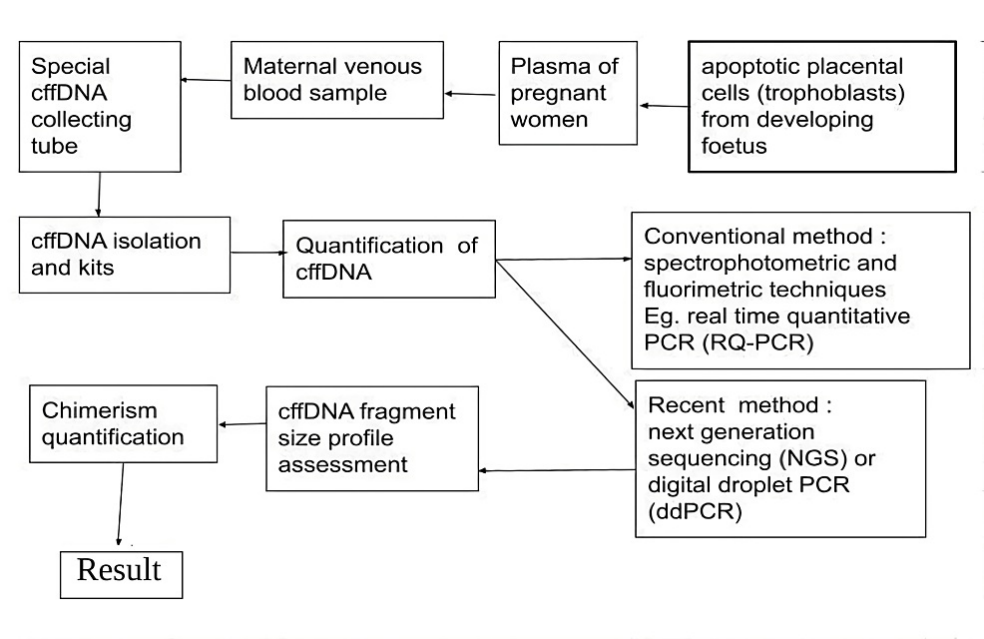
Procedure of a conventional cffDNA analysis cffDNA: Cell-free foetal DNA, PCR: Polymerase chain reaction, RQ-PCR: Real-time qualitative PCR, NSG: Next generation sequencing, ddPCR: Digital droplet PCR [20]

NIPT involves analyzing the cell-free fetal DNA (cffDNA), as shown in Figure 2, present in a maternal blood sample to determine the likelihood of fetal aneuploidy. Serum screening which also employees the maternal blood serum, remains an essential part of screening for first and second trimesters of pregnancy. NIPT is more accurate than serum screening and produces fewer false positives for many conditions The blood of the pregnant lady is taken from the vein and is collected in the specially made cffDNA blood tube. The blood is then fed in the NIPT assay, which carries out cffDNA isolation followed by its quantification. Examples of isolating kits are MagNA Pure 24 (Roche®), IDEAL (IDSolution®), LABTurbo 24 (Taigen®) and Chemagic 360 (Perkin Elmer®), while quantification method examples include digital droplet PCR (ddPCR), BIABooster system and QUBIT fluorometer. Conventionally, these two steps are performed in two different settings, which consume much time. Newer methods can isolate and quantify the cffDNA altogether at once. The analyzing machine selects a single cell of the fetus and amplifies its genomic content to form multiple copies. Now the machine looks for any variation in the number or structure of chromosomes and gives a report. 

Cell-Free Foetal DNA Analysis for Trisomies

In the last decade, numerous studies have focused on the clinical applicability of cffDNA in common trisomies and sex aneuploidies. Based on many studies, as mentioned in Table 3, it is now established that cffDNA is highly accurate for screening various trisomies, especially trisomy 21 or down syndrome, trisomy 18 or Edward syndrome, and Patau syndrome or trisomy 13. 

**Table 3:** Studies associated with cffDNA analysis for trisomies.

Article/ study	Sample size	Result
Bogaert et al. [21]	153,575 pregnant females	Positive predictive value of Down syndrome: 92.4%, Edward syndrome: 84.6%, Patau syndrome: 43.9%
Kostenko et al. [22]	101,899 pregnant females	Detection rate being Patau syndrome: 94%, Down syndrome: 100%, Edward syndrome: 97%
Verde et al. [23]	36,456 pregnant females	Sensitivity Down syndrome: 99.2%, Edward syndrome: 91.2%, Patau syndrome: 84.4%

Cell-Free Fetal DNA Analysis for Aneuploidies of Sex Chromosomes

Any abnormal distribution of chromosomes sum total, which is not a multiple of its normal haploid number, i.e., 23, due to addition or deletion of one or more chromosomes, leading to a skewed chromosome complement, is known as aneuploidies. The well-known sex chromosome aneuploidies are Turner syndrome (45, X), Klinefelter syndrome (47, XXY), 47, XXX and 47, XYY. These people suffer from various neurological and endocrinological manifestations along with fertility issues. Cell-free fetal DNA analysis has become a vital screening modality for "at risk" fetuses with reasonable accuracy, as shown in various studies mentioned in Table 4. 

**Table 4:** Studies associated with cffDNA analysis of aneuploidies SPR: Slide positivity rate, PPV: Positive predictive value, SCA: Sex chromosomal abnormalities

Article/ study	Sample size	Result
Lai et al. [24]	86,193 pregnant females with reportable results	SPR: 0.6%, PPV (SCA): 38%, i.e., n=505 Sensitivity: 45, X: 88.46%; 47, XXY: 100%; 47, XXX: 100%; 47, XYY: 100%
Lu et al. [25]	222,107 pregnant females with reportable results	PPV: 45, X: 18.14%; 47, XXY: 80.29%; 47, XXX: 58.73%; 47, XYY: 71.19%
Margiotti et al. [26]	9,985 pregnant females with reportable results	SCA Incidence: 0.31%, i.e., 31 positive cases of which 77.3% were true positive, i.e., 17 true positive cases out of which 45, X: 53%; 47, XXY: 23.5 %; 47, XXX: 17.6%; 47, XXY: 5.9%

Cell-Free Fetal DNA Analysis for Rare Trisomies

RAT or rare autosomal trisomies are chromosomal abnormalities that are infrequently seen at birth. Apart from trisomy 21, 18, and 13, the most common rare aneuploidies detected by cffDNA are chromosomes 22, 16, 15, and 7. Studies mentioned in Table 5 show that the accuracy of screening is comparable to that of CVS. Positive cffDNA tests often have been linked with an increased likelihood of pregnancy-related complexities, including stillbirth, IUGR, and fetal mosaicism. 

**Table 5:** Studies associated with cffDNA analysis for rare trisomies. RAT: Rare autosomal trisomies, CVS: Chorionic villi sampling

Article/ study	Sample size	Result
Scott et al. [27]	23,338 pregnant females with reportable results	Incidence: 1/835, i.e., n= 28; RAT was found in Trisomy 7:6; Trisomy 16: 4; Trisomy 22: 3
Pertile et al. [28]	89,817 pregnant females with reportable results	Incidence: 0.34%,, i.e., n=306; Trisomy 7: 0.0746%; Data was comparable to the CVS

Cell-Free Fetal DNA Analysis for Blood Grouping of a Fetus

Invasive prenatal procedures like cordocentesis, amniotic fluid test, and chorionic villi sampling have become obsolete in determining the growing fetus's blood group along with the Rh status. Cell-free fetal DNA analysis is a safer and more reliable method to know the blood group and Rh status of the fetus to give Rh prophylaxis in the case of RhD-negative pregnant mother carrying an RhD-positive fetus [29,30]. It is also valuable for prognosticating the fetal red cell antigen status [31].

Cell-Free DNA Analysis for Microdeletion Syndromes

A rare chromosomal disorder that results in the loss or deletion of various segments of chromosomes that are not big enough to be visualized by conventional microscopy or karyotyping. Currently, cfDNA analysis is being used to screen for the presence of a common microdeletion syndrome termed 22q11.2, which is known by multiple names like DiGeorge syndrome and velo-cardio-facial syndrome [32]. It is a syndrome with a variable presentation. In the studies by Duan et al. [33] and Kagan et al. [34], it is found that the tests exhibit moderate detection rates and are less accurate. 

Role of Failure Rate and Fetal Fraction

The fetal fraction or FF is defined as the amount of cell-free fetal DNA present in total cell-free DNA. It is a fraction between cffDNA and cfDNA. Higher the fraction, the easier it becomes to detect and distinguish fetal DNA from maternal DNA, giving a better and more accurate result. Cell-free fetal DNA starts to rise from the sixth week of pregnancy but reaches the cut-off value only by the 10^th^ week of pregnancy. After that, the cffDNA fraction goes on rising in the maternal plasma. For machines to analyze and give results, a certain fraction of cffDNA is required, below which sensitivity and specificity for the test decrease rapidly. For most companies involved in the analysis of cffDNA, 4% of total cell-free DNA is the cut-off below which a test yields no result or a failed test [35]. The DR or detection rate with only 4% cffDNA or below is 62.1%, while with ⪰9% cffDNA, DR approaches 100% [36]. Factors which can affect the cffDNA fraction in the maternal plasma are the collection of the sample is wrong, unspecialized blood tubes, mishandling of the sample while transportation and false results by the dedicated laboratories themselves. Also, the timing of the test is crucial as samples taken before 10 weeks of pregnancy are likely to yield no report due to insufficient fetal fraction. Notably, the cffDNA is on the lower side in twin pregnancies compared to singleton pregnancies [37]. The various conditions related to the fetal fraction are summarized in Table 6. 

**Table 6:** Foetal fraction and its inference. [37,38]

Foetal fraction (FF)	Possible conditions
Decreased	Increasing maternal age and basal metabolic rate, twin pregnancies (especially with dichorionic twins), Trisomy-13, Trisomy-18 and triploidy, assisted reproductive techniques.
Increased	Gestational age, Trisomy-21

Limitations of Cell-Free Fetal DNA Analysis

The foremost characteristic of a good screening test is its affordability. This factor alone can decide whether a given population will receive or undergo a particular test. In the field of prenatal testing, the emergence of more sophisticated non-invasive prenatal tests has seen a rise in the demand and price of these tests. For example, in the USA, the average price of cffDNA analysis can vary from $700-$2200 [39], which is about ₹56,000-₹176,000 in Indian Rupees. Meanwhile, Indian laboratories, which are limited in number, charge anything ranging from 10,000-25,000. So, for the lower-class population, the test seems to be out of reach in terms of affordability and availability. The occurrence of low FF or foetal fraction often leads to failed reporting of the test even though the FF was present in adequate amounts. The test is limited by its own wrong sampling and transportation methods, uncertain and outdated laboratory practices and parameters, and errors made by the technicians. 

With the ease of getting tested with the cffDNA analysis, people are often tempted to know the gender of the foetus beforehand, especially in India, where preference for male children is seen. Once the gender is accurately established, it will only facilitate discriminatory sex-selective abortion [40]. The advantage of screening a foetus as early as 10 weeks has proved critical to the parents, both positively and negatively. The results of cffDNA analysis are not always accurate as they can be false-positive or false-negative, giving parents a false sense of concern and relief, respectively. Parents should undergo pre-test and post-test genetic counselling to understand these new approaches and their results and not be burdened to act so early in the course of pregnancy upon the result of a DNA screening test that is yet to be confirmed by diagnostic means.

## Conclusions

A prenatal test that poses almost no risk to the fetus and mother, cell-free fetal DNA testing has emerged as a very promising screening test. With exceptional accuracy, its capability of screening for chromosomal abnormalities, especially Down syndrome, Patau syndrome, and Edward syndrome, has made it a screening modality of choice for the general population. With newer technologies and accurate methods, it is now possible to screen beyond the specified use of cffDNA analysis to test for rarer trisomies and sex chromosomal aneuploidies with increased sensitivity and specificity. Although each laboratory has its method of analyzing abnormal fetal DNA, which has given rise to questionable authenticity of their accuracy claims, most of the positive cffDNA test has been confirmed positive with the help of diagnostic modalities. Countries like Belgium and Netherland have adopted NIPT as a primary-tier government-funded evaluation test for their population. At the same time, many other developed nations use them as second-tier publicly funded screening tests. In India, people are reluctant to pay a hefty sum just for screening purposes. The government needs to emphasize opening standard laboratories across the country to bring down the cost and regulate the test and stop its misuse by people who get themselves tested for the primary objective of determining the gender of the fetus and opting for termination of pregnancy.
